# Supplementary material for: Changes in cerebrospinal fluid proteins across the spectrum of untreated and treated chronic HIV-1 infection
Source: PLoS Pathog. 2024 Sep 24;20(9):e1012470. doi: 10.1371/journal.ppat.1012470 (PMC11469498; doi:10.1371/journal.ppat.1012470)
Supplement: S1 Appendix — This appendix contains brief glossaries relating to the proteins identified in the figures. (DOCX) [file ppat.1012470.s002.docx]

**S1 Appendix. Glossaries of proteins featured in Main and Supplemental figures.**

These glossaries provide the names and brief descriptions of the ***Olink*** measured proteins identified by their gene name abbreviations with figure panels showing the concentrations across subject groups in **Main Figures 2-4** and **Supplemental Figure S1 and S2**. The brief descriptions are extracted from UniProt: <https://www.uniprot.org/uniprotkb/?query=*>

***Figure 2 Glossary of CSF proteins shown in Panel D with brief comments on patterns of change across groups.***

***LILRA5 (A6NI73)***. (*Leukocyte immunoglobulin-like receptor subfamily A member 5*). LILRA5 may play a role in triggering innate immune responses. The ***HAD*** group had highest concentrations, but levels in the ***NSE*** and ***2ryE*** groups were also elevated above the ***CD4-defined*** groups that exhibited a gradual increase in concentrations, i.e., the *myeloid* pattern of concentration change, with CD4+ T-cell loss.

***CD48 (P09326)***. This protein is a cell surface glycoprotein that interacts via its N-terminal immunoglobulin domain with cell surface receptors, including 2B4/CD244 or CD2, to regulate immune cell function and activation; it participates in T-cell signaling. The ***NSE*** group median CD48 concentration was higher than that of the ***HAD*** group; the ***CD4-defined*** groups exhibited the *lymphoid* concentration change pattern.

***MZB1*** (***Q8WU39***). (*Marginal zone B- and B1-cell-specific protein*). MZB1 associates with immunoglobulin M (IgM) heavy and light chains, promotes IgM assembly and secretion, and helps to diversify peripheral B-cell functions. The mean concentration of the ***NSE*** group was higher than that of the ***HAD*** group. Interestingly, the levels in the ***AsE*** group were also mildly elevated, while the ***CD4- defined*** groups showed the *lymphoid* pattern of change.

***SH2D1A*** (***O60880***): (*SH2 domain-containing protein 1A*). SH2D1A regulates receptors of the signaling lymphocytic activation molecule (SLAM) family. It also can promote CD48-, SLAMF6 -, LY9-, and SLAMF7-mediated NK cell activation. Its concentrations in the ***NSE*** group were higher than that of the ***HAD*** group and the ***CD4 defined*** groups exhibited the *lymphoid* pattern.

***PDCD1*** (***Q15116***). (*Protocadherin alpha-C1)*. PDCD1 is an Inhibitory receptor on antigen activated T-cells that plays a critical role in induction and maintenance of immune tolerance. The concentrations in the ***NSE*** group were higher than those of the ***HAD*** group, and the ***CD4-defined*** groups exhibited a shallow *lymphoid* pattern.

***ICAM3*** (***P32942***). (*Intercellular adhesion molecule 3*). ICAM3 is in family of ligands for the leukocyte adhesion protein LFA-1 (integrin alpha-L/beta-2) and also a ligand for integrin alpha-D/beta-2. In association with integrin alpha-L/beta-2, it contributes to apoptotic neutrophil phagocytosis by macrophages. The mean concentration in the ***NSE*** group was clearly higher than that of the ***HAD*** group and the ***CD4-defined*** groups exhibited a *lymphoid* pattern.

***CD27*** (P26842). (*Receptor for CD70/CD27L*). CD27 may play a role in survival of activated T-cells and in apoptosis. The median concentration in the ***NSE*** group was slightly higher than that of the ***HAD*** group, and the ***CD4-defined*** groups exhibited a *lymphoid* pattern with relatively high concentrations peaking in the ***CD4 50-199*** group at levels near those of the **HAD** group. The concentrations in the ***AsE*** group were also mildly elevated, as were half of the values in the two treatment-suppressed groups indicating some inflammatory changes persisting in these groups.

***CLEC6A*** (***Q6EIG7***). (**C-type lectin domain family 6 member A).** CLEC6A **a**cts as a pattern recognition receptor of the innate immune system, drives maturation of antigen-presenting cells and shapes antigen-specific priming of T-cells toward effector T-helper 1 and T-helper 17 cell subtype. Because nearly 40% of the measurements were below their LODs, minor protein changes in the groups with lower values might have been somewhat obscured, though the overall pattern of change across groups was similar to that of PCDC1 described above with a shallow *lymphoid* pattern in the ***CD4-defined*** groups and with ***NSE*** higher than ***HAD*** group.

***CD79B*** (***P40259***). (**B-cell antigen receptor complex-associated protein beta chain).** CD79B **is required for** initiation of the signal transduction cascade activated by the B-cell antigen receptor complex. Here the ***CD4-defined*** groups exhibited the *lymphoid* pattern with higher con**centrations in the NSE than in the HAD group.**

***BTN3A2*** (***P78410***). (**Butyrophilin subfamily 3 member A2). This protein p**lays a role in T-cell responses in the adaptive immune response and inhibits the release of interferon gamma from activated T-cells. The ***HAD*** and ***NSE*** group concentrations were nearly equal, with a *myeloid* pattern in ***CD4-defined*** groups.

***Figure 3 Glossary of CSF proteins shown in Panel B***

***CD48 (P09326)***. *This cell surface glycoprotein* interacts via its N-terminal immunoglobulin domain with cell surface receptors including 2B4/CD244 or CD2 to regulate immune cell function and activation; participates in T-cell signaling.

***TNFRSF9 (Q07011)***. (**Tumor necrosis factor receptor superfamily member 9). A r**eceptor for TNFSF9/4-1BBL that is possibly active during T-cell activation.

***ADA2 (Q9NZK5)***. (**Adenosine deaminase 2).** ADA2 **m**ay contribute to the degradation of extracellular adenosine, a signaling molecule that controls a variety of cellular responses; it may play a role in the regulation of cell proliferation and differentiation*.*

***PDCD1 (Q15116)***. (*Protocadherin alpha-C1*). PDCD1 is a potential calcium-dependenT-cell-adhesion protein. It may be involved in the establishment and maintenance of specific neuronal connections in the brain.

***SH2D1A O60880***). (*SH2 domain-containing protein 1A*). Regulates receptors of the signaling lymphocytic activation molecule (SLAM) family. Can also promote CD48-, SLAMF6 -, LY9-, and SLAMF7-mediated NK cell activation.

***IL12B (P29460)****.* (**Interleukin-12 subunit beta).** Can act as a growth factor for activated T and NK cells, enhance the lytic activity of NK/lymphokine-activated killer cells, and stimulate the production of IFN-gamma by resting PBMCs; promotes production of pro-inflammatory cytokines*.*

***MZB1 (Q8WU39)***. (*Marginal zone B- and B1-cell-specific protein*). This protein associates with immunoglobulin M (IgM) heavy and light chains, promotes IgM assembly and secretion, and helps to diversify peripheral B-cell functions.

***IL1RN (P18510)****.* (**Interleukin-1 receptor antagonist protein).** An anti-inflammatory antagonist of interleukin-1 family of proinflammatory cytokines; it protects from immune dysregulation and uncontrolled systemic inflammation triggered by IL1 for a range of innate stimulatory agents including pathogens*.*

***ICAM3*** ***(P32942)***. (*Intercellular adhesion molecule 3*)*.* A protein in the family of ligands for the leukocyte adhesion protein LFA-1 (integrin alpha-L/beta-2) and a ligand for integrin alpha-D/beta-; it contributes to apoptotic neutrophil phagocytosis by macrophages.

***FASLG (P48023)****:* (**Tumor necrosis factor ligand superfamily member 6). *FASLG* b**inds to TNFRSF6/FAS, a receptor that transduces the apoptotic signal into cells; it is involved in cytotoxic T-cell-mediated apoptosis, natural killer cell-mediated apoptosis and in T-cell development.

***CSTB (P04080)***. (**Cystatin-B). An i**ntracellular thiol proteinase inhibitor.

***GDF15 (Q99988)***. (**Growth/differentiation factor 15). A** Macrophage inhibitory cytokine; regulates food intake, energy expenditure and body weight in response to metabolic and toxin-induced stresses.

***F11R (Q9Y624)***. (**Junctional adhesion molecule A). A l**igand for integrin alpha-L/beta-2 involved in memory T-cell and neutrophil transmigration; likely plays a role in epithelial tight junction formation.

***TNFRSF12A (Q9NP84)***. (**Tumor necrosis factor receptor superfamily member 12A). A w**eak inducer of apoptosis in some cell types. It promotes angiogenesis and the proliferation of endothelial cells and may modulate cellular adhesion to matrix proteins.

***PLAUR (Q03405*)**. (**Urokinase plasminogen activator surface receptor).** Mediates the proteolysis-independent signal transduction activation effects of urokinase plasminogen activator.

***ATOX1 (O00244)***. (**Copper transport protein ATOX1).** May be important in cellular antioxidant defense*.*

***TNFRSF1B (P20333)****.* (**Tumor necrosis factor receptor superfamily member 1B). This protein m**ediates most of the metabolic effects of TNF-alpha. Isoform 2 blocks TNF-alpha-induced apoptosis, suggesting that it regulates TNF-alpha function by antagonizing its biological activity.

***TNFRSF1A (P19438)***. (*T***umor necrosis factor receptor superfamily member 1A).** Receptor for TNFSF2/TNF-alpha and homotrimeric TNFSF1/lymphotoxin-alpha. death-inducing signaling complex (DISC) performs caspase-8 proteolytic activation which initiates the subsequent cascade of caspases (aspartate-specific cysteine proteases) mediating apoptosis. Contributes to the induction of non-cytocidal TNF effects including anti-viral state and activation of the acid sphingomyelinase.

***CH3L1 (also YKL-40) (P36222)***. (**Chitinase-3-like protein 1).** Plays a role in T-helper cell type 2 (Th2) inflammatory response and IL-13-induced inflammation, inflammatory cell apoptosis, dendritic cell accumulation and M2 macrophage differentiation. May play a role in tissue remodeling. (See also **Figure S2**)

**SCARB2 (Q14108)**. (**Lysosome membrane protein 2). The protein a**cts as a lysosomal receptor for glucosylceramidase (GBA1) targeting and for enterovirus 7.

**SIGLEC1 (Q9BZZ2)**. (**Sialoadhesin).** Endocytic receptor mediating clathrin-dependent endocytosis. Macrophage-restricted adhesion molecule that mediates sialic-acid dependent binding to lymphocytes, including granulocytes, monocytes, natural killer cells, B-cells and CD8 T-cells*.*

***LILRA5 (A6NI73).*** (*Leukocyte immunoglobulin-like receptor subfamily A member 5*)*.* This protein may play a role in triggering innate immune responses.

***LGALS9 (O00182)***. (**Galectin-9).** Binding of this protein to HAVCR2 induces T-helper type 1 lymphocyte (Th1) death. This protein: stimulates bactericidal activity in infected macrophages by causing macrophage activation; is a ligand for P4HB and CD44; it promotes ability of mesenchymal stromal cells to suppress T-cell proliferation; expands regulatory T-cells and induces cytotoxic T-cell apoptosis following virus infection; and induces migration of dendritic cells; Inhibits natural killer (NK) cell function; enhances microglial TNF production.

***ADAM8 P78325***. (**Disintegrin and metalloproteinase domain-containing protein 8). This protein is** possibly involved in leukocyte extravasation.

***GRN (P28799)***. (**Progranulin). GRN is a** key **r**egulator of lysosomal function and a growth factor involved in inflammation, wound healing and cell proliferation; promotes epithelial cell proliferation by blocking TNF-mediated neutrophil activation preventing release of oxidants and proteases; modulates inflammation in neurons by preserving neurons survival, axonal outgrowth and neuronal integrity.

***BTN3A2 (P78410)***. (**Butyrophilin subfamily 3 member A2).** Plays a role in T-cell responses in the adaptive immune response. Inhibits the release of IFNG from activated T-cells.

***IL1RN (P18510)***. (**Interleukin-1 receptor antagonist protein).** Anti-inflammatory antagonist of interleukin-1 family of proinflammatory cytokines. Protects from immune dysregulation and uncontrolled systemic inflammation triggered by IL1 for a range of innate stimulatory agents such as pathogens.

***TMSB10 (P63313)***. (**Thymosin beta-10). Plays an** important role in the organization of the cytoskeleton.

***Glossary of CSF proteins shown in Figure 4, Panel E.***

***CD40L (P29965)***. (*CD40 ligand*). Acts as a ligand to CD40/TNFRSF5; co-stimulates T-cell proliferation and cytokine production.

***CD1C (P29017)***. (**T-cell surface glycoprotein CD1c). An** antigen-presenting protein that binds self and non-self lipid and glycolipid antigens and presents them to T-cell receptors on natural killer T-cells.

***TNFSF14 (O43557)***. (*Tumor necrosis factor ligand superfamily member 14*). Acts as a ligand for TNFRSF14/HVEM to deliver costimulatory signals to T-cells, leading to T-cell proliferation and IFNG production.

***CD160 (O95971)***. (**CD160 antigen). A r**eceptor on immune cells capable to deliver stimulatory or inhibitory signals that regulate cell activation and differentiation; signaling pathways via phosphoinositol 3-kinase in activated NK cells and via LCK and CD247/CD3 zeta chain in activated T-cells; receptor for both classical and non-classical MHC class I molecules; triggers NK cell cytotoxic activity, likely playing a role in anti-viral innate immune response; on CD8+ T-cells, binds HLA-A2-B2M in complex with a viral peptide and provides a costimulatory signal to activated/memory T-cells.

***CD6 (P30203)***. (*T-cell differentiation antigen CD6*). A cell adhesion molecule that mediates cell-cell contacts and regulates T-cell responses. It functions as costimulatory molecule and promotes T-cell activation and proliferation.

***CD244 (Q9BZW8)***. (*Natural killer cell receptor 2B4*)*.* A receptor of the signaling lymphocytic activation molecule (SLAM) family; its ligand is CD48. It is involved in the regulation of CD8^+^ T-cell proliferation; expressed on activated T-cells.

***CD8A (P01732)***. (**T-cell surface glycoprotein CD8 alpha chain).** CD8A **p**lays an essential role in the immune response and serves multiple functions in responses against both external and internal offenses. In T-cells, it functions primarily as a coreceptor for the MHC class I molecule:peptide complex*.*

***CRTAM (O95727).*** (**Cytotoxic and regulatory T-cell molecule).** CRTAM mediates heterophilic cell-cell adhesion which regulates the activation, differentiation and tissue retention of various T-cell subsets.

***CCL2 (P13500)***. (**C-C motif chemokine 2;** *also Monocyte chemotactic protein 1. MCP-1*)**.** CCL2 *ex*hibits chemotactic activity for monocytes; it serves as a ligand for C-C chemokine receptor CCR.

***CCL7 (P80098).*** (**C-C motif chemokine 7). A c**hemotactic factor that attracts monocytes and eosinophils.

***IL15 (P40933)***. (*Interleukin-15*). IL15 plays a major role in the development of inflammatory and protective immune responses to microbial invaders; stimulates the proliferation of natural killer cells, T-cells and B-cells and promotes the secretion of several cytokines. It induces the production of IL8 and CCL2.

***LILRA5 (A6NI73).*** (*Leukocyte immunoglobulin-like receptor subfamily A member 5*)*.* May play a role in triggering innate immune responses.

***Glossary of CSF proteins in Figure S1 with quadruplicate measurements: CXCL8, IL6 and TNF.***

***CXCL8 (P10145)***. (Interleukin-8). This is a chemotactic cytokine that mediates inflammatory response by attracting neutrophils, basophils, and T-cells. It is released in response to inflammatory stimuli, exerts its effect by binding to the G-protein-coupled receptors CXCR1 and CXCR2, and is found in neutrophils, monocytes and endothelial cells. Changes in CXCL8 concentrations across the specimen groups are modest with a shallow lymphoid pattern across the CD4-defined groups, and modestly higher concentrations in the ***NSE*** than ***HAD*** groups.

***IL6 (P05231)*** (Interleukin-6) This cytokine has a wide variety of biological functions in the innate immune response; it is synthesized by myeloid cells, including macrophages and dendritic cells, upon recognition of pathogens through toll-like receptors at the site of infection or tissue injury. In the adaptive immune response, it is required for the differentiation of B cells into immunoglobulin-secreting cells. It plays a major role in the differentiation of CD4+ T cell subsets. IL6 shows a rather flat pattern across the CD4-defined groups, modest elevation in the ***HAD*** group that is higher than the small increase in the ***NSE*** group. The highest median is in the small ***2ryE*** group.

***TNF (P01375)*** (Tumor necrosis factor, also TNF-alpha, cachectin) TNF is a multifunctional proinflammatory cytokine, mainly secreted by macrophages and involved in the regulation of a wide spectrum of biological processes. Here also the changes across the ***CD4-defined*** groups are shallow, perhaps suggesting a low-level lymphoid pattern, though as discussed above many values are below the LODs, interfering with interpretation or comparison. The **HAD** and ***NSE*** show nearly equal mild, though distinct, elevations. Highest median is in the small ***2ryE*** group.

***Glossary of CSF proteins in Figure S2, biomarkers of CNS injury measured in Olink Explore platform.***

**NEFL** ***(P07196)*** (*Neurofibrilary light chain protein*, also *NfL*). This is a major intracellular axonal protein that is shed into the CSF after neuronal-axonal injury. It has proved very useful as a CSF (and blood) biomarker of CNS injury in a number of neurodegenerative conditions including HIV-1 infection (see text).

**MAPT** *(P10636)*. (*Microtubule-associated protein*, also *tau*). CSF levels of MAPT show a more blunted response to HIV-related injury than NEFL, as previously reported [123, 156]. MAPT promotes microtubule assembly and stability in neurons and is involved in the establishment and maintenance of neuronal polarity. It forms the neurofibrillary tangles characteristic of Alzheimer’s Disease.

**APP *(P05067)***. (*Amyloid-beta precursor protein*). APP functions as a cell surface receptor and performs physiological functions on the surface of neurons relevant to neurite growth, neuronal adhesion and axonogenesis. Interaction between APP molecules on neighboring cells promotes synaptogenesis.

**GFAP *(P14136)***. (*Glial fibrillary acidic protein*). GFAP is a cell-specific intermediate filament that distinguishes astrocytes from other CNS cells.

**CHI3L1/YKL-40** ***(P36222)***. (***Chitinase-3-like protein 1*). Systemically** CHI3L1/YKL-40 plays a role in T-helper cell type 2 (Th2) inflammatory response and IL-13-induced inflammation, inflammatory cell apoptosis, dendritic cell accumulation and M2 macrophage differentiation. It has also been implicated as an astrocytic biomarker in CSF.

**TREM2** ***(Q9NZC2)***. (*Triggering receptor expressed on myeloid cells*). TREM2 acts as a receptor for amyloid-beta protein 42, a cleavage product of the amyloid-beta precursor protein, APP, and mediates its uptake and degradation by microglia.

**KYNU** ***(Q16719)***. (*Kynureninase*). This is an enzyme in L-kynurenine pathway considered to contribute to inflammation and neuronal injury.
