# Supplementary material for: Changes in cerebrospinal fluid proteins across the spectrum of untreated and treated chronic HIV-1 infection
Source: PLoS Pathog. 2024 Sep 24;20(9):e1012470. doi: 10.1371/journal.ppat.1012470 (PMC11469498; doi:10.1371/journal.ppat.1012470)

Figure S1A. Correlations of quadruplicate CXCL8 measurments.

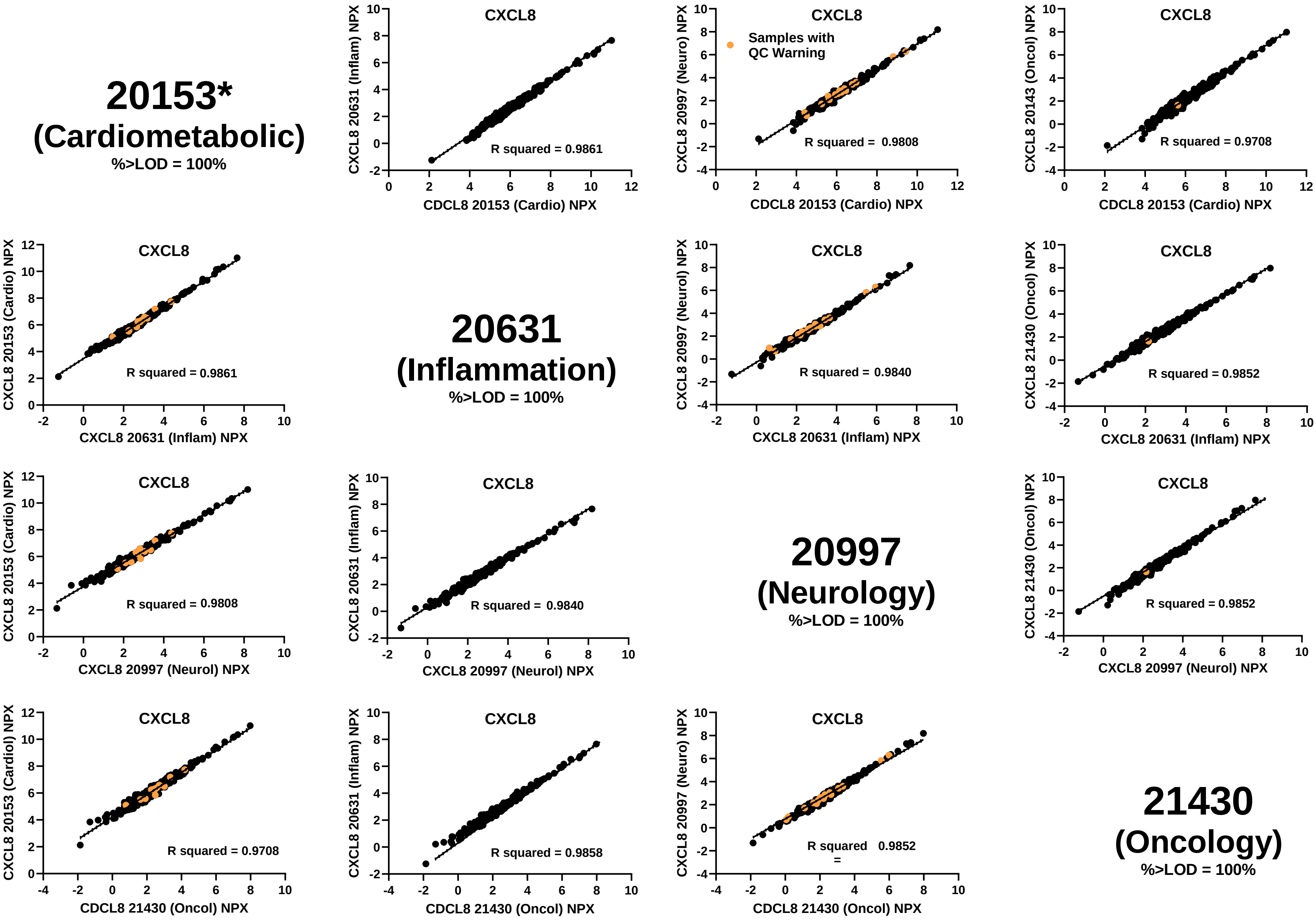

Figure S1B. Correlations of quadruplicate IL6 measurments.

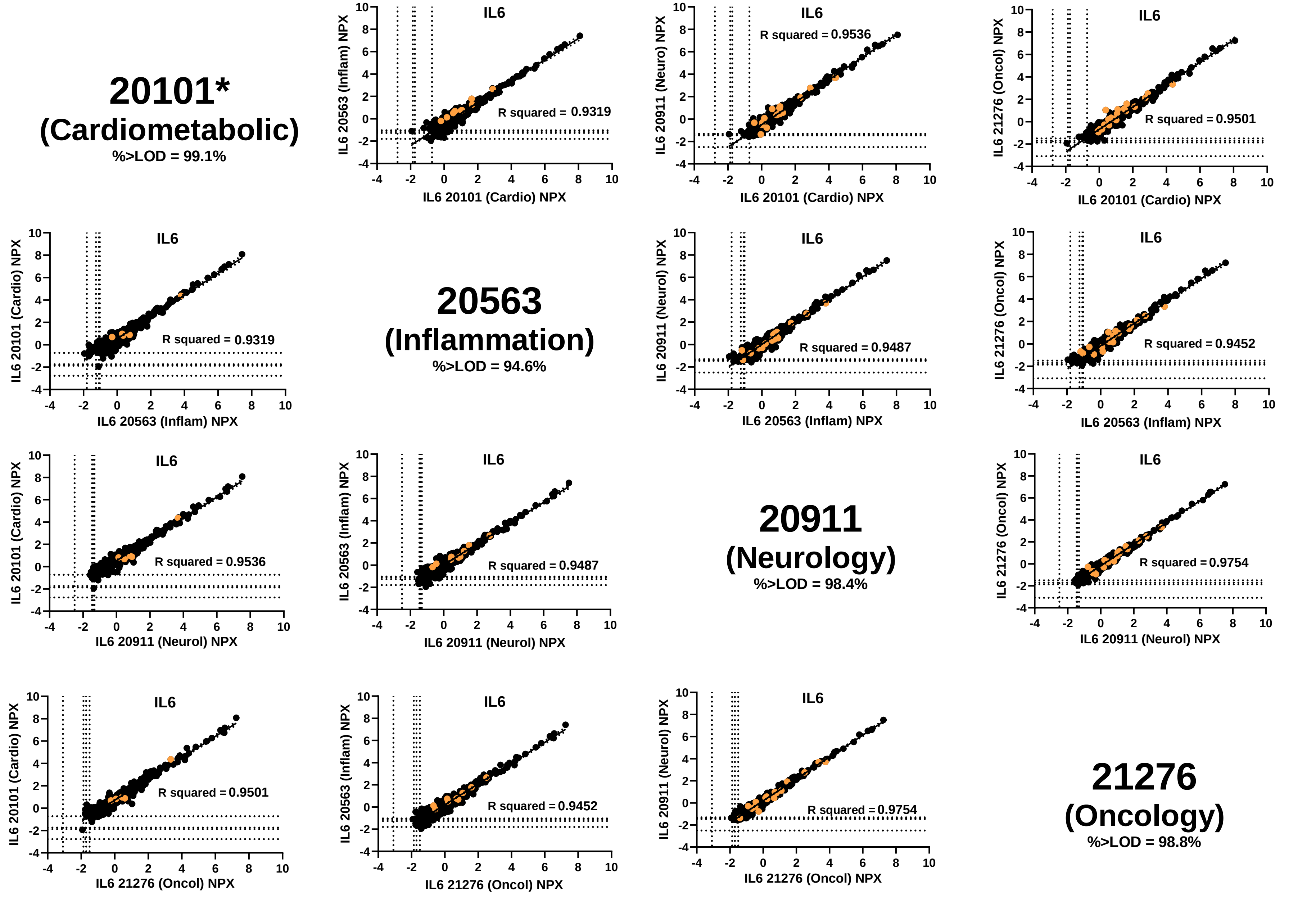

Figure S1C. Correlations of quadruplicate TNF measurments.

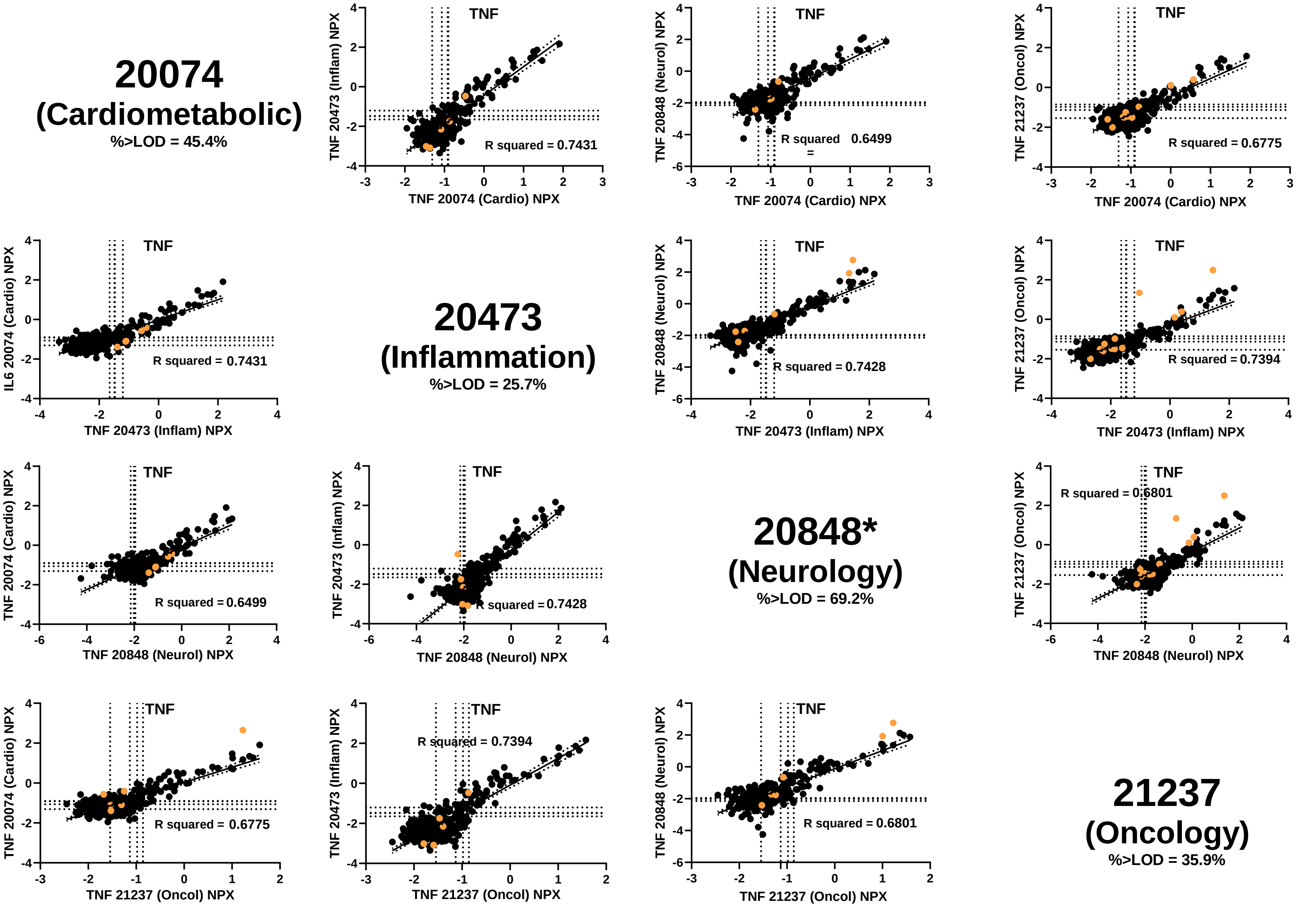

Figure S1D. Subject group profiles across quadruplicate measurements.

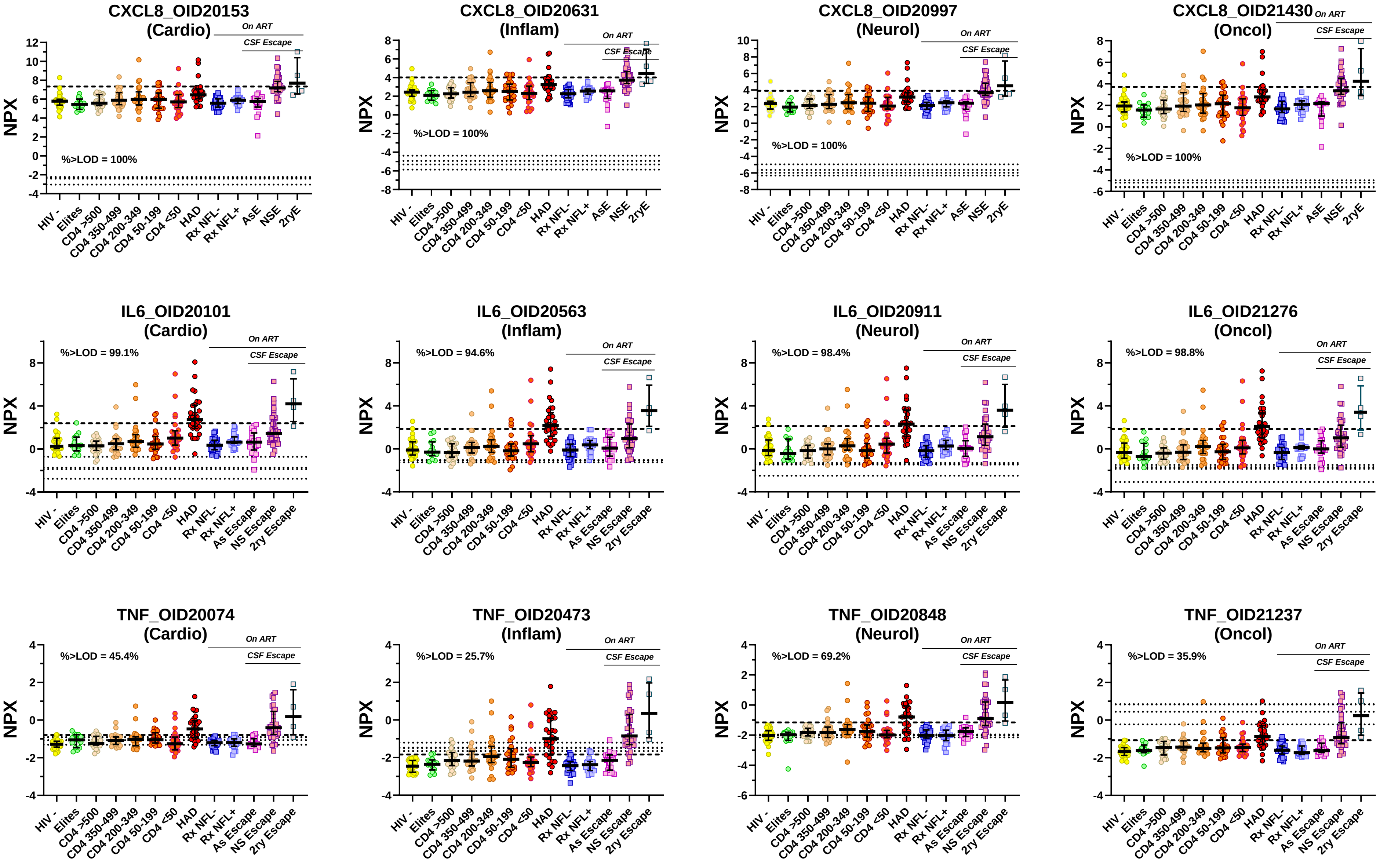

Supplement: S1 Fig — This figure examines the correlations among quadruplicate assays of three proteins and other selected features including QC warnings and LOD measurements of inflammatory molecules. The Olink Explore 1536 platform uses four individual assay plates (Cardiometabolic, Inflammation, Neurology and Oncology), each with sets of protein detection antibodies (two antibodies for each protein measured). As part of the quality control strategy, three of the proteins (CXCL8, IL6 and TNF) are measured on all four plates, i.e., in quadruplicate, using the same antibody pairs but in the context of different sets of background antibody pairs on each of these plates. S1 Fig examines the intercorrelations of the assay results and compares the patterns of study specimen profiles generated by each of the quadruplicate assays across the spectrum of the 307 study specimens (this preliminary exploration included all of the specimens, including the four miscellaneous samples omitted from the main analysis). Additionally, the plates are structured so that assay of each protein is divided into four component sets of measurements, each with a separately calculated level of detection (LOD). Examination of the three sets of quadruplicate measurements in S1 Fig provides an opportunity to concretely illustrate these methodological features which are annotated for every protein in the full data table (File 1 Dataset of CSF proteins in chronic HIV _infection_rev2024-09-10.xlsx DOI: 10.5061/dryad.x3ffbg7tv). A-C. Correlations among quadruplicate plate assay results. These panels include three montages, each with 12 graphs showing assay correlations among the quadruplicate assays with identification of the QC Warnings related to individual measurements and notations of assay LOCs. The format for the first set (CXCL8) is outlined in more detail that can then be applied to the other two sets of quadruplicates (IL6 and TNF), while the LOD notations are more prominent in the second two sets, and particular [file ppat.1012470.s003.pdf]
