# Supplementary figures and images for: Changes in cerebrospinal fluid proteins across the spectrum of untreated and treated chronic HIV-1 infection
Source: PLoS Pathog. 2024 Sep 24;20(9):e1012470. doi: 10.1371/journal.ppat.1012470 (PMC11469498; doi:10.1371/journal.ppat.1012470)

# Figure S2. CNS Injury markers.

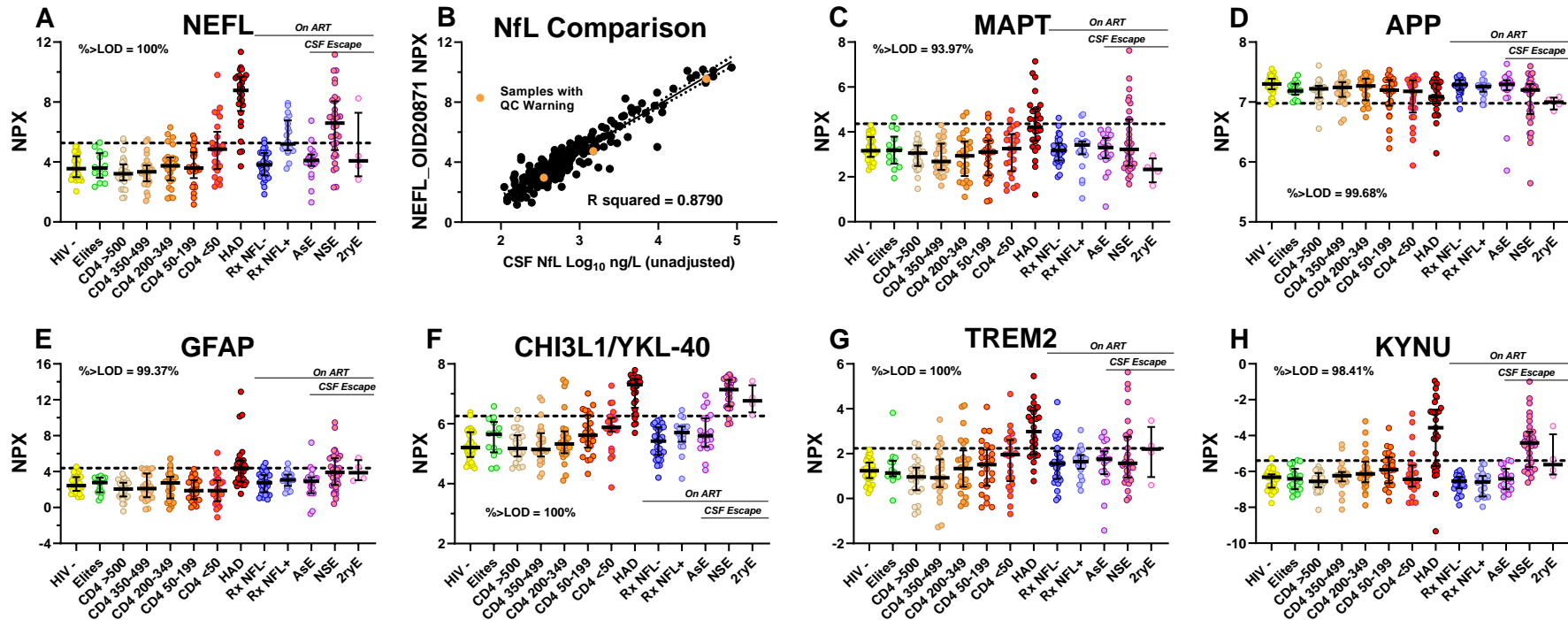

Supplement: S2 Fig — The figure shows the results of seven Olink Explore 1536 protein measurements that have been applied to assess CNS injury in other studies. In S2A and S2B-H Fig the format is the same as that for other CSF biomarker changes across the groups, including the horizontal dashed line that indicates the mean + 2 SD of the HIV- controls as a visual reference for comparison across group (or– 2 SD in the case of APP in S2D Fig in which the concentration changes in the PWH were negative). The percentage of measurement above the LODs for each of these is also listed, with all in a range that allows interpretation of the values across the subject groups. Following the nomenclature used in the Olink Explore 1536 platform, the proteins are designated by their gene names. This includes the Olink measurement of NfL. In this paper we use the abbreviation, NEFL, for the Olink results while using NfL as the abbreviation for general references to this protein and to the results of other assays, Including the UMAN ELISA used for the earlier measurements performed in the University of Gothenburg Laboratory of Neurochemistry that are presented in Fig 1O. The following sections briefly discuss each panel in the figure. A. The NEFL measurements generated in the Olink Explore 1536 exhibit a pattern of concentration changes very similar to that seen with the UMAN ELISA results presented earlier in Fig 1O. Greatest elevations are present in the HAD group, minor increases in the CD4 <50 group, and substantial elevations in the NSE group (though lower than in HAD). The Rx NFL+ group shows clear elevation with a median near the level of the HIV- mean + 2 SD dashed line. This pattern might be considered as the prototypic neuronal pattern of change across the sample group. It is similar to the myeloid pattern, perhaps distinguished by the flat concentration levels in the CD4-defined groups with blood CD4+ T cells above 50 per μL, along with elevation in the NSE. Whether the general elevation in the [file ppat.1012470.s004.pdf]
