# Supplementary material for: Changes in cerebrospinal fluid proteins across the spectrum of untreated and treated chronic HIV-1 infection
Source: PLoS Pathog. 2024 Sep 24;20(9):e1012470. doi: 10.1371/journal.ppat.1012470 (PMC11469498; doi:10.1371/journal.ppat.1012470)
Supplement: Attachment — Submitted filename: Letter to the editors on corrected resubmission 2024-07-20.docx [file ppat.1012470.s005.docx]

To the editors and reviewers of our MS, "Changes in Cerebrospinal Fluid Proteins across the Spectrum of

Untreated and Treated Chronic HIV-1 Infection” for consideration in PLOS Pathogens (PPATHOGENS-D-24-00640 - [EMID:f4d076ae92aadf3a]).

***Response to request*** for further corrections to MS: **PLOS Pathogens: Technical Check requests for PPATHOGENS-D-24-00640R1 - [EMID:d6e5949ea7711f24]**

July 20/2024

Thank you for the requests for further changes related to errors in our previous submission and for further changes related to PLOS formatting and informational requests. This letter 1) outlines our responses to the recent requests in the initial section, and 2) includes our initial revised submission letter outlining our MS revisions for the convenience of the Editors and Reviewers. All revisions are now included in this submission.

The responses to the recent request for corrections are listed in the numerical order of the request.

1. **CrediT author contributions**. *We have now listed each of the coauthor’s contributions in the order of authorship using the CrediT Role definitions after the initials of each coauthor. This is in a section following the authors’ email addresses.*
2. Amended **Financial Disclosures**. *We have edited the Financial Disclosure section text as requested to include the funding sources with the PIs’ initials with the requested detail and statement that the funders had no role in the study etc. This information is now placed at the end of the MS with the Supporting Information.*
3. **Matching Funders and Disclosures**: *The funders and grant numbers in the Funding Information should now match listings in the Financial Disclosures*. We have clarified both the Financial Disclosures and Supporting Information.
4. **Data posting**. *We still plan to post the dataset (Table S1) online on the Dryad site if the MS is accepted by PP. We plan that these data will be fully accessible. We are committed to open data as also explained in the MS text.*
5. **Repair of errors** including duplication of MS files with the *submission of revised MS files*. *We have now removed duplicate files and uploaded fresh copies of the revised MS without duplication.*
6. **Updated MS**. *We have included a new clean copy of revised/corrected MS as well as copy of with Track Changes of the revised MS.*
7. **Title of submission** and MS have been reconciled. Both are now: ***Changes in Cerebrospinal Fluid Proteins across the Spectrum of Untreated and Treated Chronic HIV-1 Infection***. I am not certain how numbers were inserted withing the title at submission, but likely were picked up from the line numbering on the submission. We have corrected this for the resubmission and in the title of this submission above.
8. **Author lists**: the Author List in MS matches list in online submission form.
9. As above. We have **included Sarah Beth Joseph** who was inadvertently omitted in the ONLINE submission form.
10. The **trademark symbol** found associated with NF-light assay has been removed. We are unaware of any other such symbols elsewhere.
11. The full list of legends for the **Supporting Information** file is included after the References. This includes *listings of the Supplemental Tables (Table S1 and Table S2), Supplemental Figures (Fig S1 and Fig S2 and Appendix S1*. The files for these materials are uploaded with the Supporting Information.
12. We have also replaced the four **Main Figures** with *versions that have been processed through PACE*.

Thank you again for your assistance in meeting these requirements. I hope we have fully addressed each point satisfactorily (but I will not be shocked if there are some persistent or new errors detected). We very much appreciate your patience and assistance in getting to a fully compliant version of the MS for review. If further errors are identified or have been introduced with the recent edits, we apologize in advance.

***Letter Submitted with initial MS revision:***

July 14/2024

To the editors and reviewers of our MS, "Changes in Cerebrospinal Fluid Proteins 1 across the Spectrum of 2 Untreated and Treated Chronic HIV-1 Infection" for consideration in PLOS Pathogens (PPATHOGENS-D-24-00640 - [EMID:f4d076ae92aadf3a]).

*We very much appreciate the reviewers’ comments and now submit a revised MS which we think addresses the issues that they have raised. These revisions have improved the manuscript and clarified some points that enhance its accessibility and utility for the reader.*

*In undertaking this revision, we first addressed the comments and suggestions of the two Reviewers and then made additional changes. These included moving the* **MATERIALS AND METHODS** *section to before the* **RESULTS AND DISCUSSION** *section and undertaking additional text edits to further enhance clarity that indirectly also addresses the Reviewers’ comments and our own refreshed re-reading of the MS after a break. We think this is now an improved, clearer document, though the data, including figures and tables are largely unchanged (though Table S1 has been updated with further detail). The conclusions are also similar to those expressed previously, though hopefully a bit clearer. We have also rearranged and enhanced parts to conform to the formal requirements of PLOSPathogens formatting. This rearrangement and editing may make following our point-by-point responses to the specific suggestions a bit more difficult, but we assure you that they have all been carefully addressed.*

*In addition to including the Reviewers’ comments below before explaining some of the individual manuscript changes, the entire original letter containing the Editor and Reviewers comments is included within this letter. As requested, we have uploaded two versions of the revised MS: 1. a clean final revision, and 2. A version of this same draft that documents and annotates our revisions in detail, along with the annotations provided by activation of the Track Changes feature though with our extensive revisions beyond those responding to the reviewers, it may be a bit dense. Below we provide a further point-by-point discussion of our initial responses to the two Reviewers’ comments.*

*We have made the following general formatting and organizational changes:*

*The MS is now double spaced with attention to consistency of the (only) 3 levels of Headings along with other features of PP formatting instructions.*

*We have added an* ***Author Summary****.*

*We have reorganized the sections to place the* **MATERIALS AND METHODS** *before the* **RESULTS AND DISCUSSION** *sections. We think this will help the reader and avoid some of the issues with study group definitions and other similar problems encountered by the Reviewers. As instructed, we have uploaded two versions of the revised MS, one with track changes feature activated to annotate revisions and a clean copy with all of these changes accepted.*

*The figures, unchanged from the previous submission, will now have been processed according to the PLOS guidelines and resubmitted with this response. The Legends to the four Main Figures are embedded in the MS, but for clarity we have enclosed these in boxes to set them apart.*

*We have also moved the Supplemental material (including* ***Table S2****,* ***Figures S1 – S2*** *and the* ***Glossaries of Gene-Protein Functions*** *for* ***Figures 2 – 4****) to the* **SUPPORTING INFORMATION** *section placed after the* ***References****.*

*We have reduced redundancies between the* **RESULTS AND DISCUSSION** *text and the* ***Legends*** *for* ***Figures 2 – 4*** *and placed these Legends along with that of* ***Figure 1*** *directly under the place where these figures will appear in the text, following the PLOS guidelines. In general, we have rearranged parts of the MS to further reduce redundancies and increase clarity and ease for the reader and reviewer.*

*If this MS is accepted, even provisionally, we will upload* ***Table S1*** *containing the full study dataset to Dryad (*[*https://datadryad.org/stash*](https://datadryad.org/stashxxxx)*).*

*Before taking up the individual responses, we would like to comment on the reviewers’ requests regarding specific clinically-defined group comparisons (e.g., elite controllers vs treated-suppressed individuals) that were not included in this MS. It is our overall intent to pursue this and other targeted group comparisons in future analyses and MSS based on these data. They were omitted in this initial submission because it is already quite long. The one comparison included in this MS, comparison of HAD and NSE groups, was included because of the particular neurological interest of these two groups and as a prototype and example of the potential utility of such comparisons. However, the main focus of the current MS was on providing an introduction to and an overview of the full data set. This provides a starting point for additional explorations. Overall, the rich data introduced in this MS will require such additional analyses to address questions with a more targeted approach. We plan to follow with some of these focused analyses but also have urged others to independently use these data (now available online) to join in this undertaking. In this commentary we use the group abbreviations employed in the MS.*

*In the following response, we have placed the Reviewer’s comments in* black plain typeface *and our responses in blue italic text. We begin by responding to the General Comments of the two Reviewers.*

GENERAL COMMENTS

Reviewer #1: The authors used proteomic profiling to characterize 303 CSF specimens from 12 groups of participants from 4 clinical sites. The signatures identified confirmed two previous patterns observed in previous studies using only a few markers including the lymphoid (inflammatory changes that increase with decreasing CD4 count until decreasing when below 50 cells/uL) and myeloid (increasing levels with deceasing CD4 count). Antiretroviral treatment mostly mitigated the changes but not necessarily to control levels. The authors have generously made the data fully available for others to collaborate. It would be helpful to also have some of the key neurocognitive scales and reservoir measures to further contextualize the findings. Additional considerations are included here.

*Our response: This is a fair (and generous) brief overview of some of the main general findings. With respect to the neurocognitive scales and reservoir measurements, the study used archived samples— predominantly from two centers (Gothenburg and San Francisco) supplemented by samples from two other centers (Milan and Pune) because of their particular neurological interest (HAD and NSE groups). We also used clinical diagnoses that included the assessments of study-related physicians in categorizing individual participants and patients. The individual volunteers and patients were not consistently assessed with quantitative neuropsychological testing, so we did not include cognitive test scores in the analysis or list these tests in the full data sheet (Table S1). Additionally, while test scores are useful in some settings, they can also be misleading related to: a) variations in background social, economic and cultural factors, and b) the confounding effects of previous HIV-related and non-HIV-related CNS insults leaving legacy impairments. In dealing with biomarker correlations our emphasis has been on the comparison of analysis during a single time point within the group category. While it would be valuable to analyze longitudinal observations, that is another study. The same is true for assessments of the CNS reservoir. However, a number of these individuals have been or are being studied with additional virological measures which will allow cross reference between publications with use of common coded subject IDs. We have now introduced some of the above omissions and caveats in the* ***Methods*** *and the* ***Limitations of the study section of the Results-Discussion*** *sections of the MS.*

Reviewer #2: This is a comprehensive, thoughtful study of cerebrospinal fluid (archived specimens) protein expression in 303 patients with HIV (PWH) that include ART-treated and untreated PWH through mild and severe neurocognitive impairment (HIV-associated dementia). Uninfected control patient samples were also included, as were samples from patients with neurosymptomatic and asymptomatic CSF escape (NSE, ASE). This is a non-biased sampling of more than 1400 proteins, and this study is unique in its relatively large size and its detailed information on the clinical and functional status of the PWH. Additionally, the examination of NSE is a unique feature. It is rigorously performed. The response to ART adds yet another important piece of information to this comprehensive study.
The authors acknowledge controversies in the field, and they thoughtfully frame points worthy of more investigation and discussion, some of which may be ignored by researchers and clinicians. The background information is clearly presented, up-to-date and comprehensive. They acknowledge the limitation of previous studies (including their own), and they appropriately emphasize the limitations of associations of markers with clinical status and pathways as not distinguishing between driving or contributing to pathogenesis or rather responding to pathogenic processes. The conclusions are strongly supported, and they are not over-reaching.
The findings of responses associated with lymphoid and/or myeloid lineages is novel, and strongly consistent with the entry and replication of lymphocyte-tropic, non-M-tropic strains into the CNS with subsequent evolution of M-tropism, establishment of a myeloid reservoir and possibly a lymphoid reservoir (?meninges). The association of CSF Nfl levels with a myeloid pattern of marker expression and not with CSF vRNA levels (which associate with lymphoid marker pattern) is an enlightening observation. Also HAD indeed associates with a myeloid pattern while NSE associates with a lymphoid pattern. These observations and interpretations are consistent with our knowledge of the entry into and evolution within the CNS of HIV strains. Overall, this study greatly enhances our knowledge and potential understanding of the CNS infection process and consequences thereof.

*Our response: This (also generous) paragraph emphasizes and nicely summarizes some of the main points of the MS. It is followed by some specific questions and suggestions which are discussed below.*

Minor points:

1) It would be helpful to speculate more about the role of CSF or plasma Nfl quantification in judging the degree of neuronal injury in less severely impaired patients. It appears that it may not really be useful. How helpful is it in cases other than HAD? As it is now clinically available, should plasm Nfl be routinely monitored in PWH? In selected patients?

*Our response: We have expanded the discussion of NfL measurement in the Introduction (in paragraph beginning on line 155), though not fully addressing these questions. In general, both CSF and blood NfL correlate with disease severity, but the latter also depends on the length of time of active injury and background of the individua. We do not discuss this interesting issue further because it was not specifically addressed by the study. Rather we emphasized the relation to other biomarkers collected within the same sample.*

2) The study involves analyses of CSF proteins, as have many smaller studies previously. Some comment about other pathogenic moieties (lipid peroxidation products, ROS, mtDNA, metabolites, others) that might also be relevant to pathogenesis can further help the reader to appreciate the complexity of neuropathogenesis.

*Our response: We briefly allude to non-protein molecules in neuropathogenesis at the end of the Results and Discussion section (line 711).*

3) Figure 2 includes data from HIV- and elite controllers----this is defined appropriately, but it should probably be introduced in the background section. Are there enough data to speculate about distinctions between elite controllers and virally-suppressed PWH?

*Our response: We have added the definition we used for the elite controllers in the text (line 744). There are likely sufficient data to compare the inflammatory responses in the elite controllers to the virally suppressed PWHs and we fully agree that this is an interesting issue. However, this is one of those questions that we have elected not to include in this MS related to the length of the MS and in order to develop a more comprehensive comparison of the aviremic/virally suppressed groups (HIV-, elites, suppressed with normal and elevated CSF NfL) in a future dedicated analysis and MS.*

**Major Issues: Key Experiments Required for Acceptance**
Please use this section to detail the key new experiments or modifications of existing experiments that should be absolutely required to validate study conclusions.

Generally, there should be no more than 3 such required experiments or major modifications for a "Major Revision" recommendation. If more than 3 experiments are necessary to validate the study conclusions, then you are encouraged to recommend "Reject".

We first listed the new lines related to the line-by-line comments, but with further rearrangements, several of these were displaced so we now only describe the actions we took to address each of the specific comments.

Reviewer #1:

1. Line 125 – the authors should explain if the patterns of “CSF inflammatory reactions”, which are compartmentalized and evolve independently, appear different from those in the blood.

*Our response: We have added further discussion of the issue of the relationship between CNS (CSF) and systemic (blood) inflammatory responses. In this study we measured proteins only in the CSF (and not in the blood) because CSF protein biomarkers reflect the local aggregate CNS inflammatory and cellular response milieu, which is our main interest. This decision was predicated on previous studies of CSF proteins and other biomarkers that have shown both divergent and parallel protein changes that support local production of these proteins within the CSF as their main source across clinical groups. These changes in the CNS proteins across this clinical spectrum was the main focus of our study. Confining measurement to CSF simplified analysis and allowed us to clearly focus on changes within the CNS that both reflect and impact the latter’s integrity. Importantly, it also allowed the study to be financially feasible.*

2. Line 128 – the authors should clarify if the phrase “augmented by additional inflammatory biomarkers” meant that the additional markers had the same patterns but were higher or just had the same patterns.

*Our response: We have qualified this statement for clarity.*

3. Line 165 (now 172) – I would clarify that this relationship is potentially bidirectional.

*Our response: Our statement involves a simple speculation. We feel that a more elaborate discussion of CNS and systemic reservoirs may somewhat burden the narrative so we would like to leave the limited mention as is. The MS itself does not provide direct evidence pro or con regarding a CNS viral reservoir.*

4. Line 183 – since these are cross-sectional assessments, the authors should comment as to whether the AsE phenotype could potentially be an earlier version of the NSE phenotype.

*Our response: We have commented that the answer to this question is not clear and that further studies bearing on this question are needed.*

5. Line 195 (now 203) – similar to the point above the authors should state if there are instances where any participants sampled as an AsE or higher CD4 group belonged to the NSE or lower CD4 group at a later time, respectively.

*Our response: Here in this* ***Introduction*** *we now introduce the overall number of samples and subjects included in the study. We mention that 5 individuals were studied twice, before treatment and while on therapy (these are flagged by color background in Table S1). These repeat samples and other details are described later in the MS in the* ***Materials and*** ***Methods*** *and* ***Results and Discussion*** *sections of the MS where the definitions and features of the clinical groups are enumerated in more detail.*

6. Methods – it is not clear where individuals with asymptomatic neurocognitive impairment and mild neurocognitive disorder would appear with or without escape or whether there would be individuals without any impairment but had escape.

*Our response: Except for the individuals in the HAD, NSE and 2ryE groups,* *none of the PWH or controls exhibited symptoms or signs of active or progressive neurological impairment based on clinical history and physical examinations at the time of their visit. They were all characterized as ‘neuroasymptomatic’ or exhibiting only chronic stable impairments or disabilities. We did not attempt to segregate those with asymptomatic or mild neurocognitive impairment, ANI or MND, but rather classified the subjects using the broad biological and diagnostic categories provided by the group designations. We have now added this point to the group descriptions in the* ***Results and Discussion*** *section .*

7. Line 248– it is not clear if any of the CD4 category participants could have phenotypic overlap with the NSE or HAD participants.

*Our response: We have now clarified this by inserting ‘neurologically asymptomatic’ to the CD4-defined groups and we have emphasized this later in the* ***Methods****.*

8. Line 263 – the authors should explain why sCD14, as a monocyte marker, follows the pattern associated with lymphocyte reduction.

*Our response: This is an excellent point. We have added further discussion on this finding in our previous study and provided an additional reference related to sCD14 and NfL correlations in CSF in HIV infection. The downside of this and other insertions is that they may unduly expand the text and distract from the essential focus of the data and analysis.*

9. Line 291 – the authors should explain why the CSF:blood ratio is different between NSE/2ryE vs HAD if neopterin is only produced in the CNS.

*Our response: Neopterin is produced both systemically and within the CNS with their concentrations changing independently though at times in parallel. We have edited this section to try to clarify this point, but also to emphasize that CSF neopterin is mainly produced within the CNS compartment (while blood neopterin likely mainly reflects systemic production of this pteridine).*

10. Line 345 (now line 379) – the authors should describe how the LOD levels were imputed.

*Our response: These were not imputed but for each protein four LODs were provided in the data reporting from the Olink assay. The methodology for the LOD is briefly described in the* ***Methods*** *section (beginning on original line 7167) and in more detail on the website as referenced in this same paragraph. The four LOD values for each protein (one for each plate measuring the protein) are all listed in Table S2, and many of these are shown in the graphical results of the protein measurement. We used these reported LOD values to calculate the proportion of measurements above the LOD on the related plate. We alluded to this earlier and directed attention to the Methods section.*

11. Line 413– the separation was less than I would have imagined and would have thought that HAD would be the lowest; the authors should address this.

*Our response: We have added a few sentences at the end of this paragraph that may afford at least a partial explanation for the overlap in the subjects from the different groups.*

12. Line 431 – the authors should address if any of these patterns suggest a mechanistic difference with these two phenotypes (HAD and NSE).

*Our response: This issue is taken up later in the MS in the discussing protein differences between HAD and NSE.*

13. Line 486– it is not clear why log of the HIV VL or some kind of weighted sum was used versus a straight sum of these two measures.

*Our response: The sum represented a very simple measure of the two correlations in this descriptive consideration of these correlations. It also predicts the graphical depiction in Panel C of Figure 3 in the right upper corner of the proteins that correlate with both CSF NEFL and HIV RNA as discussed later in this section.*

14. Line 637 – the CD4 threshold criteria should be described.

*Our response: We have inserted a sentence explaining the rationale for the smaller CD4 range of the lowest brackets, 50-199 and <50 CD4+ T-cells per µL. Overall, these brackets were selected on the basis of previous studies of a number of CSF biomarkers including inflammatory and neural injury markers.*

15. Line 642– the HIV plasma viral load and ART use criteria should be described.

*Our response: We have specified that the CD4-defined groups were untreated (either naïve to treatment – most – or off ART for at least 6 months).*

Reviewer #2: No additional experiments needed

**Part III – Minor Issues: Editorial and Data Presentation Modifications**
Please use this section for editorial suggestions as well as relatively minor modifications of existing data that would enhance clarity.

Reviewer #1: 1. Line 105– I would consider changing to the more common “R5 viruses” instead of “M-tropic viruses”.

*Our response: Because HIV tropism depends, not on the R5 receptor but the abundance of CD4 receptors (denser on CD4 T cells than macrophages), we prefer the more biologically meaningful and contemporary terms of T-tropic and M-tropic viruses. This biological property can be (and has been) assessed in vitro using an assay in the Swanstrom laboratory. R5-expressing viruses may be either T- or M-tropic.*

*Thus, wild type (WT) HIV-1 uses a high density of CD4 for entry (as is found on T cells) and uses CCR5 as a coreceptor, and this WT R5 T-tropic virus is distinct from the evolutionary variant that has evolved to use a low density of CD4 as is found on myeloid cells, often called macrophage- or M-tropic. To use just R5 could mix WT R5 T-tropic and M-tropic viruses. We were specifically trying to make a point about M-tropic virus as distinct from R5 T-tropic virus, and we think the sentence, correctly read, does that.*

2. Line 110 – I would consider changing “morbidities” to “co-morbidities”.

*Our response: We have made this change.*

3. Line 114 – would change “T cell” to “T-cells”.

*Our response: We have made this change here and throughout the MS.*

4. Line 118– “…this ‘quadratic’ pattern of CSF viral load change…”, please clarify what this change is as it is not described or mentioned earlier.

*Our response: This is based on our earlier study which included modeling of the biomarker trends with falling CD4+ T-cells. We have explained this in the text.*

5. Line 242– the authors state that the CD4 groups were introduced earlier but a definition was not previously given.

*Our response: This is correct and, in part, a result of placing the* ***Materials and Methods*** *after the* ***Results and Discussion*** *section and is clarified by the overall rearrangement of these sections..*

6. Line 273 – the authors should mention which group was greater between NSE and 2ryE.

*Our response: The median CSF HIV RNA of the three CSF escape groups are now included in the text.*

7. Line 276 – RxNFL+ and RxNFL- should be defined.

*Our response: Thank you. We now have clarified here the definitions of these two groups.*

8. Line 291 – the AsE group is not described.

*Our response: We have now elaborated on the AsE group.*

9. Line 304 – the authors should speculate as to why the RxNFL+ group has an elevated NfL.

*Our response: We have now briefly commented on this group in several places, and introduced our plan to look more thoroughly at this issue in the future.*

10. Line 310 – should clarify if the relative concentrations are relative to HIV negative.

*Our response: By this we mean relative to each other, but not in a standard quantitative measure such as mg/mL or the like. We have added further explanation/clarification of this.*

11. Line 314 – would explain why a random selection was done rather than average of the results.

*Our response: This was something of an arbitrary choice, but our rationale centered on keeping these measurements the same as the others, as well as related to the differences of scales in some of the independent measurements of some of these three proteins (see Figure S1). We have not edited the text further here.*

12. Line 317– explain whether the LOD is irrespective of HIV negative levels or after adjustment.

*Our response: The LODs methodology is described in the Materials and Methods section of the MS and is provided in the Olink data report for each*

13. Line 367) –may need to add “of NfL” to “…whether the Olink measurement of NfL, which…”.

*Our response: In handling the two assays of the same protein, we have tried to consistently use NEFL for the Olink measurement and NfL for the protein and for results of its measurement by more standard EIA.*

14. Line 371 – would also add “age” in front of second “adjusted” at the end of the sentence. which…”.

*Our response: We have added this clarification.*

15. Line 472 – perhaps the “>” should be “<”.

*Our response: You are correct. Thank you for picking up this error. Now fixed.*

16. Line 624 – would avoid “HIV infected” to refer to people or groups per the community request and in Line 680 (now 807) change “subject” to participant.

*Our response: A problem with substituting PLWH is that these individuals were all* ***chronically*** *infected (as opposed to acute or early infection) which requires emphasis. We think it is proper to describe them as is in this spot. We have changed ‘subject’ to ‘participant’ as suggested.*

17. Line 659– it should be clarified if NSE impairment was mild, moderate or severe.

*Our response: We have indicated that all had moderate to severe CNS involvement.*

Reviewer #2: Minor points:

1) It would be helpful to speculate more about the role of CSF or plasma Nfl quantification in judging the degree of neuronal injury in less severely impaired patients. It appears that it may not really be useful. How helpful is it in cases other than HAD? As it is now clinically available, should plasm Nfl be routinely monitored in PWH? In selected patients?

*Our response: We have inserted a few additional sentences in the Introduction bearing on this issue in the paragraph beginning on line 151.*

2) The study involves analyses of CSF proteins, as have many smaller studies previously. Some comment about other pathogenic moieties (lipid peroxidation products, ROS, mtDNA, metabolites, others) that might also be relevant to pathogenesis can further help the reader to appreciate the complexity of neuropathogenesis.

*Our response: We have inserted a phrase in the Conclusions paragraph alluding to exploration of non-protein molecules.*

3) Figure 2 includes data from HIV- and elite controllers----this is defined appropriately, but it should probably be introduced in the background section. Are there enough data to speculate about distinctions between elite controllers and virally-suppressed PWH?

*Our response: We agree that this is an interesting question and we intend to examine this in the future as a focused issue, but chose not to include it in this MS. We have now added a comment on this.*

PLOS authors have the option to publish the peer review history of their article ([what does this mean?](https://urldefense.com/v3/__https:/journals.plos.org/plospathogens/s/editorial-and-peer-review-process*loc-peer-review-history__;Iw!!LQC6Cpwp!vxItKh5hkQpLCM3-yoJkC5npJDY_dWUpJkU9PvbNDUgxdU0pg_jtNma4kokyJu17eb70uQ47nUnIZRjYjHYl$)). If published, this will include your full peer review and any attached files.


*Further issues raised by both reviewers are raised in their individual point-by-point comments.*

Thank you once again for the thoughtful reviews and the opportunity to respond. We hope that the MS is now suitable for publication in PLOS Pathogens.

Sincerely,

For the authors,

Richard W Price, M.D.

Professor Emeritus

Neurology, UCSF/SFGH

UCSF/SFGH

Email: [richard.price@ucsf.edu](mailto:richard.price@ucsf.edu)

Cell: 415-310-2029

**From:** em.ppathogens.0.8b9778.48b766e6@editorialmanager.com <em.ppathogens.0.8b9778.48b766e6@editorialmanager.com> **On Behalf Of** PLOS Pathogens
**Sent:** Friday, May 24, 2024 8:50 AM
**To:** Price, Richard W <richard.price@ucsf.edu>
**Subject:** PLOS Pathogens: Decision on your submission PPATHOGENS-D-24-00640 - [EMID:f4d076ae92aadf3a]

CC: rkoup@ mail. nih. gov, gsilves@ emory. edu, "Zicheng Hu" hzc363@ gmail. com, "Paola Cinque" cinque. paola@ hsr. it, "Ameet Dravid" ameet. dravid@ gmail. com, "Lars Hagberg" lars. hagberg@ medfak. gu. se, "Aylin Yilmaz" aylin. yilmaz@ gu. se, "Henrik Zetterberg"

ZjQcmQRYFpfptBannerStart

**This Message Is From an External Sender**

This message came from outside your organization.

ZjQcmQRYFpfptBannerEnd

CC: [rkoup@mail.nih.gov](mailto:rkoup@mail.nih.gov), [gsilves@emory.edu](mailto:gsilves@emory.edu), "Zicheng Hu" [hzc363@gmail.com](mailto:hzc363@gmail.com), "Paola Cinque" [cinque.paola@hsr.it](mailto:cinque.paola@hsr.it), "Ameet Dravid" [ameet.dravid@gmail.com](mailto:ameet.dravid@gmail.com), "Lars Hagberg" [lars.hagberg@medfak.gu.se](mailto:lars.hagberg@medfak.gu.se), "Aylin Yilmaz" [aylin.yilmaz@gu.se](mailto:aylin.yilmaz@gu.se), "Henrik Zetterberg" [henrik.zetterberg@clinchem.gu.se](mailto:henrik.zetterberg@clinchem.gu.se), "Dietmar Fuchs" [dietmar.fuchs@i-med.ac.at](mailto:dietmar.fuchs@i-med.ac.at), "Johanna Gostner" [johanna.gostner@i-med.ac.at](mailto:johanna.gostner@i-med.ac.at), "Kaj Blennow" [kaj.blennow@neuro.gu.se](mailto:kaj.blennow@neuro.gu.se), "Serena S. Spudich" [serena.spudich@yale.edu](mailto:serena.spudich@yale.edu), "Laura Kincer" [laura_kincer@med.unc.edu](mailto:laura_kincer@med.unc.edu), "Shuntai Zhou" [shuntaiz@email.unc.edu](mailto:shuntaiz@email.unc.edu), "Ronald Swanstrom" [ron_swanstrom@med.unc.edu](mailto:ron_swanstrom@med.unc.edu), "Magnus Gisslén" [magnus.gisslen@infect.gu.se](mailto:magnus.gisslen@infect.gu.se)

Dear Dr. Price,

Thank you very much for submitting your manuscript "Changes in Cerebrospinal Fluid Proteins 1 across the Spectrum of 2 Untreated and Treated Chronic HIV-1 Infection" PPATHOGENS-D-24-00640 - [EMID:f4d076ae92aadf3a]for consideration at PLOS Pathogens. As with all papers reviewed by the journal, your manuscript was reviewed by members of the editorial board and by several independent reviewers. In light of the reviews (below this email), we would like to invite the resubmission of a significantly-revised version that takes into account the reviewers' comments.

We cannot make any decision about publication until we have seen the revised manuscript and your response to the reviewers' comments. Your revised manuscript is also likely to be sent to reviewers for further evaluation.

When you are ready to resubmit, please upload the following:

[1] A letter containing a detailed list of your responses to the review comments and a description of the changes you have made in the manuscript. Please note while forming your response, if your article is accepted, you may have the opportunity to make the peer review history publicly available. The record will include editor decision letters (with reviews) and your responses to reviewer comments. If eligible, we will contact you to opt in or out.

[2] Two versions of the revised manuscript: one with either highlights or tracked changes denoting where the text has been changed; the other a clean version (uploaded as the manuscript file).

Important additional instructions are given below your reviewer comments.

Please prepare and submit your revised manuscript within 60 days. If you anticipate any delay, please let us know the expected resubmission date by replying to this email. Please note that revised manuscripts received after the 60-day due date may require evaluation and peer review similar to newly submitted manuscripts.

Thank you again for your submission. We hope that our editorial process has been constructive so far, and we welcome your feedback at any time. Please don't hesitate to contact us if you have any questions or comments.

Sincerely,

Guido Silvestri
Academic Editor
PLOS Pathogens

Richard Koup
Section Editor
PLOS Pathogens

Michael Malim
Editor-in-Chief
PLOS Pathogens
orcid.org/0000-0002-7699-2064

***********************


Reviewer's Responses to Questions

**Part I - Summary**
Please use this section to discuss strengths/weaknesses of study, novelty/significance, general execution and scholarship.

Reviewer #1: The authors used proteomic profiling to characterize 303 CSF specimens from 12 groups of participants from 4 clinical sites. The signatures identified confirmed two previous patterns observed in previous studies using only a few markers including the lymphoid (inflammatory changes that increase with decreasing CD4 count until decreasing when below 50 cells/uL) and myeloid (increasing levels with deceasing CD4 count). Antiretroviral treatment mostly mitigated the changes but not necessarily to control levels. The authors have generously made the data fully available for others to collaborate. It would be helpful to also have some of the key neurocognitive scales and reservoir measures to further contextualize the findings. Additional considerations are included here.

Reviewer #2: This is a comprehensive, thoughtful study of cerebrospinal fluid (archived specimens) protein expression in 303 patients with HIV (PWH) that include ART-treated and untreated PWH through mild and severe neurocognitive impairment (HIV-assosciated dementia). Uninfected control patient samples were also included, as were samples from patients with neurosymptomatic and asymptomatic CSF escape (NSE, ASE) . This is a non-biased sampling of more than 1400 proteins, and this study is unique in its relatively large size and its detailed information on the clinical and functional status of the PWH. Additionally, the examination of NSE is a unique feature. It is rigorously performed. The response to ART adds yet another important piece of information to this comprehensive study.
The authors acknowledge controversies in the field, and they thoughtfully frame points worthy of more investigation and discussion, some of which may be ignored by researchers and clinicians. The background information is clearly presented, up-to-date and comprehensive. They acknowledge the limitation of previous studies (including their own), and they appropriately emphasize the limitations of associations of markers with clinical status and pathways as not distinguishing between driving or contributing to pathogenesis or rather responding to pathogenic processes. The conclusions are strongly supported, and they are not over-reaching.
The findings of responses associated with lymphoid and/or myeloid lineages is novel, and strongly consistent with the entry and replication of lymphocyte-tropic, non-M-tropic strains into the CNS with subsequent evolution of M-tropism, establishment of a myeloid reservoir and possibly a lymphoid reservoir (?meninges). The association of CSF Nfl levels with a myeloid pattern of marker expression and not with CSF vRNA levels (which associate with lymphoid marker pattern) is an enlightening observation. Also HAD indeed associates with a myeloid pattern while NSE associates with a lymphoid pattern. These observations and interpretations are consistent with our knowledge of the entry into and evolution within the CNS of HIV strains. Overall, this study greatly enhances our knowledge and potential understanding of the CNS infection process and consequences thereof.

Minor points:

1) It would be helpful to speculate more about the role of CSF or plasma Nfl quantification in judging the degree of neuronal injury in less severely impaired patients. It appears that it may not really be useful. How helpful is it in cases other than HAD? As it is now clinically available, should plasm Nfl be routinely monitored in PWH? In selected patients?

2) The study involves analyses of CSF proteins, as have many smaller studies previously. Some comment about other pathogenic moieties (lipid peroxidation products, ROS, mtDNA, metabolites, others) that might also be relevant to pathogenesis can further help the reader to appreciate the complexity of neuropathogenesis.

3) Figure 2 includes data from HIV- and elite controllers----this is defined appropriately, but it should probably be introduced in the background section. Are there enough data to speculate about distinctions between elite controllers and virally-suppressed PWH?

**Part II – Major Issues: Key Experiments Required for Acceptance**
Please use this section to detail the key new experiments or modifications of existing experiments that should be absolutely required to validate study conclusions.

Generally, there should be no more than 3 such required experiments or major modifications for a "Major Revision" recommendation. If more than 3 experiments are necessary to validate the study conclusions, then you are encouraged to recommend "Reject".

Reviewer #1:

1. Line 125 – the authors should explain if the patterns of “CSF inflammatory reactions”, which are compartmentalized and evolve independently, appear different from those in the blood.
2. Line 128 – the authors should clarify if the phrase “augmented by additional inflammatory biomarkers” meant that the additional markers had the same patterns but were higher or just had the same patterns.
3. Line 165 – I would clarify that this relationship is potentially bidirectional.
4. Line 183 – since these are cross-sectional assessments, the authors should comment as to whether the AsE phenotype could potentially be an earlier version of the NSE phenotype
5. Line 195 – similar to the point above the authors should state if there are instances where any participants sampled as an AsE or higher CD4 group belonged to the NSE or lower CD4 group at a later time, respectively.
6. Methods – it is not clear where individuals with asymptomatic neurocognitive impairment and mild neurocognitive disorder would appear with or without escape or whether there would be individuals without any impairment but had escape.
7. Line 248 – it is not clear if any of the CD4 category participants could have phenotypic overlap with the NSE or HAD participants.
8. Line 263 – the authors should explain why sCD14, as a monocyte marker, follows the pattern associated with lymphocyte reduction.
9. Line 291 – the authors should explain why the CSF:blood ratio is different between NSE/2ryE vs HAD if neopterin is only produced in the CNS.
10. Line 345 – the authors should describe how the LOD levels were imputed.
11. Line 413 – the separation was less than I would have imagined and would have thought that HAD would be the lowest; the authors should address this.
12. Line 431 – the authors should address if any of these patterns suggest a mechanistic difference with these two phenotypes (HAD and NSE).
13. Line 486 – it is not clear why log of the HIV VL or some kind of weighted sum was used versus a straight sum of these two measures.
14. Line 637 – the CD4 threshold criteria should be described.
15. Line 642 – the HIV plasma viral load and ART use criteria should be described.

Reviewer #2: No additional experiments needed

**Part III – Minor Issues: Editorial and Data Presentation Modifications**
Please use this section for editorial suggestions as well as relatively minor modifications of existing data that would enhance clarity.

Reviewer #1: 1. Line 105 – I would consider changing to the more common “R5 viruses” instead of “M-tropic viruses”.
2. Line 110 – I would consider changing “morbidities” to “co-morbidities”.
3. Line 114 – would change “T cell” to “T-cells”.
4. Line 118 – “…this ‘quadratic’ pattern of CSF viral load change…”, please clarify what this change is as it is not described or mentioned earlier.
5. Line 242 – the authors state that the CD4 groups were introduced earlier but a definition was not previously given (appears later on line 248).
6. Line 273 – the authors should mention which group was greater between NSE and 2ryE.
7. Line 276 – RxNFL+ and RxNFL- should be defined.
8. Line 291 – the AsE group is not described.
9. Line 304 – the authors should speculate as to why the RxNFL+ group has an elevated NfL
10. Line 310 – should clarify if the relative concentrations are relative to HIV negative.
11. Line 314 – would explain why a random selection was done rather than average of the results.
12. Line 317 – explain whether the LOD is irrespective of HIV negative levels or after adjustment.
13. Line 367 – may need to add “of NfL” to “…whether the Olink measurement of NfL, which…”.
14. Line 371 – would also add “age” in front of second “adjusted” at the end of the sentence.
15. Line 472 – perhaps the “>” should be “<”.
16. Line 624 – would avoid “HIV infected” to refer to people or groups per the community request and in Line 680 change “subject” to participant.
17. Line 659 – it should be clarified if NSE impairment was mild, moderate or severe.

Reviewer #2: Minor points:

1) It would be helpful to speculate more about the role of CSF or plasma Nfl quantification in judging the degree of neuronal injury in less severely impaired patients. It appears that it may not really be useful. How helpful is it in cases other than HAD? As it is now clinically available, should plasm Nfl be routinely monitored in PWH? In selected patients?

2) The study involves analyses of CSF proteins, as have many smaller studies previously. Some comment about other pathogenic moieties (lipid peroxidation products, ROS, mtDNA, metabolites, others) that might also be relevant to pathogenesis can further help the reader to appreciate the complexity of neuropathogenesis.

3) Figure 2 includes data from HIV- and elite controllers----this is defined appropriately, but it should probably be introduced in the background section. Are there enough data to speculate about distinctions between elite controllers and virally-suppressed PWH?

PLOS authors have the option to publish the peer review history of their article ([what does this mean?](https://urldefense.com/v3/__https:/journals.plos.org/plospathogens/s/editorial-and-peer-review-process*loc-peer-review-history__;Iw!!LQC6Cpwp!vxItKh5hkQpLCM3-yoJkC5npJDY_dWUpJkU9PvbNDUgxdU0pg_jtNma4kokyJu17eb70uQ47nUnIZRjYjHYl$)). If published, this will include your full peer review and any attached files.


If you choose “no”, your identity will remain anonymous but your review may still be made public.


**Do you want your identity to be public for this peer review?** For information about this choice, including consent withdrawal, please see our [Privacy Policy](https://urldefense.com/v3/__https:/www.plos.org/privacy-policy__;!!LQC6Cpwp!vxItKh5hkQpLCM3-yoJkC5npJDY_dWUpJkU9PvbNDUgxdU0pg_jtNma4kokyJu17eb70uQ47nUnIZaJAJX3l$).

Reviewer #1: No

Reviewer #2: No

***Further comments***:

In addition to the changes annotated above, we have performed several minor edits to enhance clarity. None of these changes are substantative, so we have not enumerated them with further comments beyond those provided by *Track Changes*.

Figure Files:

While revising your submission, please upload your figure files to the Preflight Analysis and Conversion Engine (PACE) digital diagnostic tool, [https://pacev2.apexcovantage.com](https://urldefense.com/v3/__https:/pacev2.apexcovantage.com/__;!!LQC6Cpwp!vxItKh5hkQpLCM3-yoJkC5npJDY_dWUpJkU9PvbNDUgxdU0pg_jtNma4kokyJu17eb70uQ47nUnIZQzsyo_X$). PACE helps ensure that figures meet PLOS requirements. To use PACE, you must first register as a user. Then, login and navigate to the UPLOAD tab, where you will find detailed instructions on how to use the tool. If you encounter any issues or have any questions when using PACE, please email us at [figures@plos.org](mailto:figures@plos.org).

Data Requirements:

Please note that, as a condition of publication, PLOS' data policy requires that you make available all data used to draw the conclusions outlined in your manuscript. Data must be deposited in an appropriate repository, included within the bordy of the manuscript, or uploaded as supporting information. This includes all numerical values that were used to generate graphs, histograms etc.. For an example see here on PLOS Biology: [http://www.plosbiology.org/article/info%3Adoi%2F10.1371%2Fjournal.pbio.1001908#s5](https://urldefense.com/v3/__http:/www.plosbiology.org/article/info*3Adoi*2F10.1371*2Fjournal.pbio.1001908*s5__;JSUlIw!!LQC6Cpwp!vxItKh5hkQpLCM3-yoJkC5npJDY_dWUpJkU9PvbNDUgxdU0pg_jtNma4kokyJu17eb70uQ47nUnIZRE_b3yA$).

Reproducibility:

To enhance the reproducibility of your results, we recommend that you deposit your laboratory protocols in protocols.io, where a protocol can be assigned its own identifier (DOI) such that it can be cited independently in the future. Additionally, PLOS ONE offers an option to publish peer-reviewed clinical study protocols. Read more information on sharing protocols at [https://plos.org/protocols?utm_medium=editorial-email&utm_source=authorletters&utm_campaign=protocols](https://urldefense.com/v3/__https:/plos.org/protocols?utm_medium=editorial-email&utm_source=authorletters&utm_campaign=protocols__;!!LQC6Cpwp!vxItKh5hkQpLCM3-yoJkC5npJDY_dWUpJkU9PvbNDUgxdU0pg_jtNma4kokyJu17eb70uQ47nUnIZTINk15G$)

*In compliance with data protection regulations, you may request that we remove your personal registration details at any time.* [*(Remove my information/details)*](https://urldefense.com/v3/__https:/www.editorialmanager.com/ppathogens/login.asp?a=r__;!!LQC6Cpwp!vxItKh5hkQpLCM3-yoJkC5npJDY_dWUpJkU9PvbNDUgxdU0pg_jtNma4kokyJu17eb70uQ47nUnIZaNB6fYL$)*. Please contact the publication office if you have any questions.*
